# Comparison of protective effects of nicotinamide mononucleotide and nicotinamide riboside on DNA damage induced by cisplatin in HeLa cells

**DOI:** 10.1016/j.bbrep.2024.101655

**Published:** 2024-02-06

**Authors:** Shuting Qiu, Shihan Shao, Yunheng Zhang, Yingying Zhang, Jie Yin, Yu Hong, Jun Yang, Xiaohua Tan, Chunhong Di

**Affiliations:** a Affiliated Hospital, Hangzhou Normal University, Hangzhou, China; b School of Public Health, Hangzhou Normal University, Hangzhou, China

**Keywords:** Nicotinamide mononucleotide, Nicotinamide riboside, DNA damage, Cisplatin, Reactive oxygen species

## Abstract

**Background:**

Previous studies have shown that the nicotinamide adenine dinucleotide (NAD^+^) precursors, nicotinamide mononucleotide (NMN) and nicotinamide riboside (NR), protect against endogenously or exogenously induced DNA damage. However, whether the two compounds have the same or different efficacy against DNA damage is not clear. In the current study, we systematically compared the effects of NMN and NR on cisplatin-induced DNA damage in HeLa cells.

**Methods:**

To evaluate the protective effects of NMN or NR, HeLa cells were pretreated with different doses of NMN or NR followed with 10 μM of cisplatin treatment. Cell viability was examined by Trypan blue staining assay. For observing the DNA damage repair process, HeLa cells were treated with 10 μM of cisplatin for 12 h, followed with 10 mM NMN or NR treatment for another 8, 16, 24, or 32 h, DNA damage levels were assessed for each time point by immunofluorescent staining against phosphor-H2AX (γH2AX) and alkaline comet assay. The effects of NMN and NR on intracellular NAD^+^ and reactive oxygen species (ROS) levels were also determined.

**Results:**

NMN and NR treatment alone did not have any significant effects on cell viability, however, both can protect HeLa cells from cisplatin-induced decrease of cell viability with similar efficacy in a dose-dependent manner. On the other hand, while both can reduce the DNA damage levels in cisplatin-treated cells, NR exhibited better protective effect. However, both appeared to boost the DNA damage repair process with similar efficacy. NMN or NR treatment alone could increase cellular NAD^+^ levels, and both can reverse cisplatin-induced decrease of NAD^+^ levels. Finally, while neither NMN nor NR affected cellular ROS levels, both inhibited cisplatin-induced increase of ROS with no significant difference between them.

**Conclusion:**

NR have a better protective effect against cisplatin-induced DNA damage than NMN.

## Highlights

• NR pretreatment exhibiting stronger protective effects on cisplatin-induced DNA damage than that of NMN.
• Both NMN and NR reduced cisplatin induced DNA damage in a dose-dependent manner.
• NMN and NR have similar effects in promoting the repair of damaged DNA induced by cisplatin.

## 1 Introduction

Continuously occurring DNA damage in a living organism can impact health and modulate disease states [1,2], while the DNA damage response (DDR) dutifully protects the integrity of DNA by either removing or tolerating the damage [3,4], thus ensuring the overall survival of the organism. Nicotinamide adenine dinucleotide (NAD^+^) is a co-substrate for NAD^+^ consuming enzymes, including poly ADP-ribose polymerases (PARPs) and Sirtuins (Sirt), which are important players in DDR [5,6]. Interestingly, it has been reported that NAD^+^ levels decrease while DNA damage levels increase during aging [7]. Therefore, methods for restoring NAD^+^ levels have been explored, such as supplementation of NAD^+^ precursors nicotinamide mononucleotide (NMN) and nicotinamide riboside (NR), which have been verified as an effective measure to combat DNA damage and improve certain disease states of aging, such as Alzheimer's disease (AD) in mice [8,9]. Currently, NMN and NR are marketed as food supplements in the United States and other countries that are claimed to improve glucose control, enhance energy metabolism, and reverse metabolic complications associated with aging. To date, there are over 20 clinical trials using NMN, and over 30 using NR, respectively, to evaluate their potential in anti-aging or other diseases. Therefore, it is important to have a clear understanding of the functions and underlying molecular mechanisms for these NAD^+^ precursors.

There are three pathways for the biosynthesis of NAD^+^ in cells, namely, the *de novo* biosynthesis pathway, Preiss-Handler pathway, and salvage pathway [10,11]. The salvage pathway is the primary source of NAD^+^ in mammalian cells, in which nicotinamide (NAM) is converted to NMN under the catalysis of nicotinamide phosphoribosyltransferase (NAMPT), the key rate-limiting enzyme in mammalian NAD^+^ biosynthesis; NMN is then converted to NAD^+^ by NMN adenylyltransferases (NMNATs) [12]. On the other hand, NR can be converted into NMN by nicotinamide riboside kinase (NRK), indicating that NMN is a more direct precursor for NAD^+^. NMN and NR are natural compounds that effectively enhance NAD^+^ biosynthesis which bypasses the rate-limiting enzyme NAMPT [13]. Several studies have shown that exogenous NR is transported into cells through equilibrative nucleoside transporters (ENTs), while exogenous NMN has to be converted into NR by CD38 and then transported into cells via ENTs, thus making NMN a less effective precursor for NAD^+^ *in vivo* [14]. Nonetheless, Ito et al. identified SLC12A8 as a specific NMN transporter for NMN uptake in mice [15], thus avoiding the step to be converted to NR and transported into cells by ENTs. Furthermore, it was shown that SLC12A8 in the lateral hypothalamus is important in maintaining energy metabolism and skeletal muscle functions during aging in mice [15]. Therefore, such data suggested that NMN and NR might have similar efficacy for their anti-aging effects. However, this finding has been challenged by other group [16]. Thus, it is important to compare the functions of different NAD^+^ precursors to achieve best clinical application potential.

Although accumulating evidence has demonstrated that both NMN and NR supplementation can reduce DNA damage in the aging process or other diseases [9,17,18], however, to date, no comparative study of the efficacy of these two compounds on protecting cells from DNA damage has been conducted. Therefore, in this study, we investigated whether there is any difference between NMN and NR in alleviating DNA damage. As reported here, the two compounds have similar effects on enhancing cell viability, replenishing intracellular NAD^+^ and scavenging intracellular ROS induced by cisplatin in HeLa cells. Interestingly, we found that NR might have a better protective (preventive) effect against cisplatin-induced DNA damage than NMN.

## 2 Materials and methods

### 2.1 Cell line and chemicals

HeLa cells were grown in Dulbecco modified Eagle medium (DMEM) supplemented with 10 % (v/v) of FBS, 2 mMol/L glutamine, 1 mMol/L sodium pyruvate, 100 U/mL penicillin and 100 mg/mL streptomycin at 37 °C under 5 % CO_2_. NMN (#GC16971), NR (#GC44401), and cisplatin (#GC11908) were purchased from GlpBio Company (Montclair, CA, USA).

### 2.2 Cell survival assay

Cell viability was measured by Trypan blue exclusion assay as described before [19]. After cells were harvested and re-suspended with fresh medium, 20 μL cell suspension and 20 μL 0.4 % trypan blue solution were mixed, and then 20 μL mixture was transferred to a hemocytometer. Live cells excluded trypan blue dye, whereas dead cells were stained, and they were counted under a microscope. The cell viability (%) was calculated as: (number of live cells/number of total cells) × 100 %. Experiments were performed in triplicates.

### 2.3 Immunofluorescent staining of γH2AX

The expression of **γ**H2AX was evaluated as described before with modifications [19,20]. First, cells were fixed in 4 % paraformaldehyde for 30 min, and then permeabilized in 0.1 % Triton-X 100 and immersed three times for 5 min each in a washing solution of PBSTX (0.1 %) (phosphate buffer saline 0.1 mol/L, pH 7.5, 0.1 % Triton X-100) at room temperature. The slides were incubated with a blocking solution of PBSTX (0.1 %) containing 1 % bovine serum albumin (BSA) for 60 min at room temperature. Slides were then rinsed with PBS three times for 5 min each and incubated with γH2AX antibody (Cat# ab81299, Abcam) solution in a moisture chamber for 16 h at 4 °C. After washing with PBS, a secondary goat-*anti*-rabbit IgG antibody conjugated with Alexa Fluor 633 was added and incubated for 2 h. Slides were then rinsed in PBS three times and the nucleus was stained with DAPI at a concentration of 0.1 μmol/L for 10 min at room temperature. Images were acquired on a Zeiss LSM 710 single-photon confocal system using a multitrack configuration. The intensity of immunofluorescence was measured by ImageJ software.

### 2.4 Alkaline comet assay

Comet assay was conducted as described before with some modifications [21]. In short, 75 μL of pre-warmed regular melting point agarose (0.7 %) at 70 °C was used as the first gel layer. 10 μL of cells (approximately 1 × 10^5^) were mixed with 75 μL of pre-warmed low melting point agarose (0.7 %) at 37 °C. 10 μL of these mixtures were used as the second gel layer. After solidification, the cometslides were dipped into a cold lysis solution (1 % Triton X-100, 10 % dimethyl sulfoxide and 89 % lysis buffer containing 10 mmol/L Tris, 2.5 mol/L NaCl and 100 mmol/L Na_2_EDTA, pH 10) for an hour. After lysis, cometslides were placed in a horizontal electrophoresis chamber filled with cold alkaline buffer and incubated for 20 min in the dark for DNA unwinding, and then electrophoresis was performed (20 V, 20 min). For neutralization, cometslides were washed in PBS for 10 min. After air-drying, cometslides were stained with GelRed for scoring. Tail length was scored using ImageJ in 100 randomly selected nuclei per sample.

### 2.5 Measurement of NAD ^+^ levels

NAD^+^ was measured using the NAD^+^/NADH Assay Kit following the manufacturer's instructions (Beyotime, Shanghai, China). Briefly, cells were plated in six-well dishes at a density of 3 × 10^5^ cells/well overnight. After various treatments, cells were washed 3 times in ice-cold PBS and were extracted in 150 μL extraction buffer. 20 μL lysates were added to a 96-well plate and mixed thoroughly with an ethanol dehydrogenase working solution, and incubated at 37 °C for 10 min the chromogenic solution was then added to the plate and the mixture was further incubated at 37 °C for 30 min. The absorbance was measured at 450 nm and analyzed using SPARK microplate multimode reader. The total concentration of NAD^+^ in the cell samples was calculated according to the standard curve. All measurements were repeated at least thrice in independent experiments.

### 2.6 Measurement of reactive oxygen species (ROS) levels

ROS was measured using the ROS Assay Kit (Beyotime, Shanghai, China). Briefly, 3 × 10^5^ HeLa cells were seeded in 6-well plates overnight. After various treatments, cells were washed twice with PBS and incubated with ROS detection reagent (DCFH-DA, 5 μmol/L) at 37 °C for 30 min. Subsequently, the cells were washed thrice with PBS. Finally, the fluorescence signal was detected using SPARK microplate multimode reader.

### 2.7 Statistical analysis

Each experiment was repeated at least three times independently. Data were presented as Mean ± SD. Two-way ANOVA Tukey's multiple comparisons test was applied to confirm the significant differences between the groups. Results were considered statistically significant with *P* < 0.05. The statistical evaluation between different groups was analyzed using the R software.

## 3 Results

### 3.1 NMN and NR protect HeLa cells from cisplatin-induced decrease of cell viability in a dose-dependent manner

**Fig. 1:**
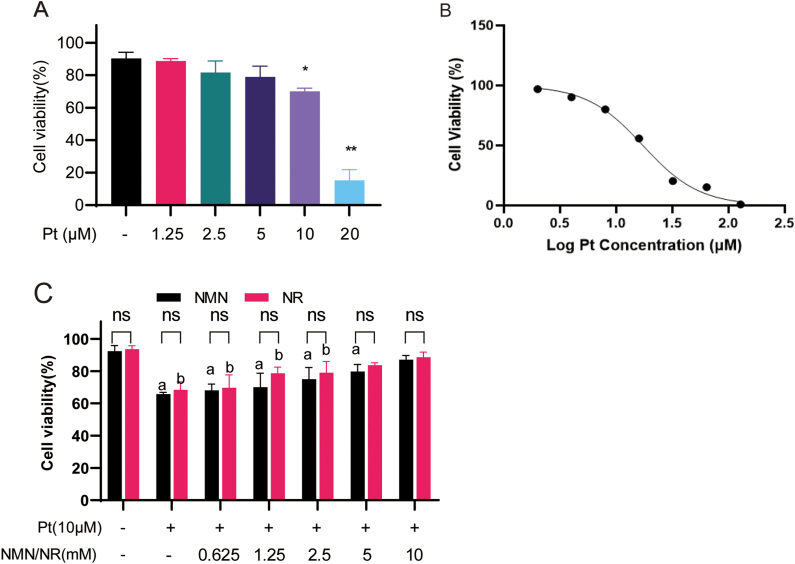
NMN and NR rescue cell viability of cisplatin-treated cells. (A) HeLa cells were treated with different concentrations of cisplatin (Pt) for 24 h and then the cell viability was measured by trypan blue exclusion assay. *: P < 0.05; **: P < 0.01, compared to control group (−). (B) The dose-response curve of cisplatin and cell viability. (C) Cell viability of HeLa cells co-treated with different concentrations of NMN or NR and 10 μmol/L cisplatin. a, *P* < 0.05, compared to the control group (−); b, *P* < 0.05, compared to Pt group. (For interpretation of the references to colour in this figure legend, the reader is referred to the Web version of this article.)

Firstly, the cytotoxic effect of cisplatin on HeLa cells was evaluated. HeLa cells were treated with different doses of cisplatin (0, 1.25, 2.5, 5, 10, and 20 μmol/L) for 24 h, then the cell viability was determined by Trypan blue exclusion assay. As shown in Fig. 1A, cisplatin decreased cell viability in a dose-dependent manner, and an IC50 value of 17 μmol/L was obtained (Fig. 1B). In subsequent experiments, 10 μmol/L cisplatin was chosen, because the cell viability was significantly reduced at this dose, but enough cells could still be collected.

Then, the effects of NMN and NR on the viability of cisplatin-treated cells were examined. HeLa cells were pre-treated with different concentrations (0, 0.625, 1.25, 2.5, 5, and 10 mmol/L) of NMN or NR and then 10 μmol/L cisplatin for 12 h. The results showed that both NMN and NR enhanced the cell viabilities in a dose-dependent manner compared with cisplatin-treated cells (P < 0.05, Fig. 1C), but there was no significant difference in cell viability between NMN and NR cotreatment groups.

### 3.2 NR has a better protective effect against cisplatin-induced DNA damage than NMN

**Fig. 2:**
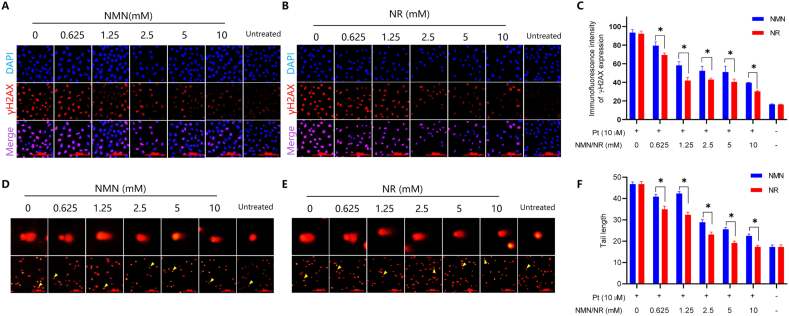
NR exhibits a better protective effect against cisplatin-induced DNA damage than NMN. Hela cells were pretreated with indicated concentrations of NMN or NR for 12 h, and then exposed to 10 μmol/L cisplatin (Pt) for another 12 h, the cells were harvested and the DNA damage levels were assayed. (A–B) Cells were fixed and stained with anti-γH2AX antibody and subjected to immunofluorescent microscopy. Shown were representative images from one of three independent experiments. (C) Quantitation of γH2AX fluorescent intensity of (A) and (B) by ImageJ software. *P < 0.05. (D–E) Representative images of alkaline comet assay of HeLa cells treated as above. Shown were from one of three independent experiments. The yellow arrow indicated the cell in the enlarged image. (F) The tail length of the comet assay was quantified and analyzed by ImageJ software. *P < 0.05. (For interpretation of the references to colour in this figure legend, the reader is referred to the Web version of this article.)

To examine the protective effects of NMN or NR on DNA damage, HeLa cells were incubated with 0.625, 1.25, 2.5, 5, and 10 mmol/L NMN or NR for 12 h, then the cells were exposed to 10 μmol/L cisplatin with fresh medium for another 12 h. The γH2AX immunofluorescent assay results showed that pretreatment with NMN or NR decreased the γH2AX levels in a dose-dependent manner (Fig. 2A and B). The intensity of fluorescence was quantified by ImageJ software. At each dose, NR-treated cells had relatively lower γH2AX levels than NMN-treated cells (*P* < 0.05, Fig. 2C). The alkaline comet assay is another assay to evaluate DNA damage level through tail length for fragmented DNA after electrophoresis. Similarly, the tail lengths were shortened in NMN- or NR-pretreated cells in a dose-dependent manner (Fig. 2D and E), and the NR-pretreated groups showed even shorter tail lengths than NMN-pretreated groups (*P* < 0.05; Fig. 2F). These results indicated that both NMN and NR mitigated cisplatin-induced DNA damage in a dose-dependent manner, whereas NR had a stronger protective effect against DNA damage induced by cisplatin than NMN.

### 3.3 NMN and NR have similar effects in promoting the repair of cisplatin-induced DNA damage

DDR senses DNA damage and initiates repair mechanisms to maintain genomic stability. We then compared the effects of NMN and NR on the repair of damaged DNA. HeLa cells were exposed to 10 μmol/L cisplatin for 12 h, and then cultured in fresh medium with 10 mmol/L NMN or NR. Hereafter, the cells were harvested every 8 h. The DNA damage levels were assessed.

**Fig. 3:**
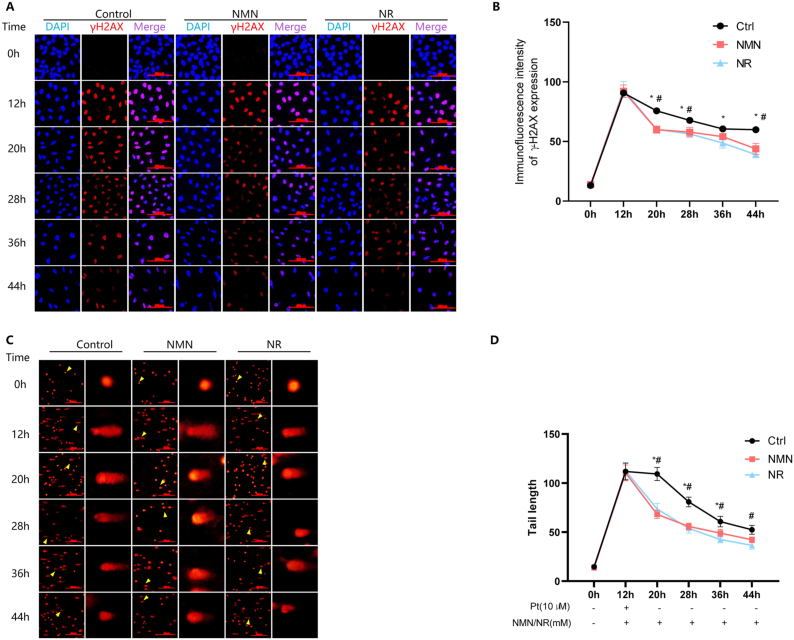
NMN and NR promote the repair of cisplatin-treated DNA damage. HeLa cells were exposed to 10 μmol/L cisplatin (Pt) for 12 h, and then the cells were cultured in fresh medium with the addition of 10 mmol/L NMN or NR, and the cells were harvested every 8 h until 44 h. (A) Cells were fixed and stained with anti-γH2AX antibody and subjected to immunofluorescent microscopy. Shown were representative images from one of three independent experiments. (B) Quantitation of γH2AX fluorescent intensity by ImageJ software. *, *Ρ* < 0.05, compared to NMN treatment; #, *P* < 0.05, compared to NR treatment. (C) Shown were representative images of alkaline comet assay of HeLa cells treated as above from one of three independent experiments. The yellow arrow indicated the cell in the enlarged image. (D) The tail length of comet assay was quantified and analyzed by ImageJ software. *, *P* < 0.05, compared to NMN treatment; #, *Ρ* < 0.05, compared to NR treatment. (For interpretation of the references to colour in this figure legend, the reader is referred to the Web version of this article.)

After cisplatin was removed, the fluorescent intensity of γH2AX decreased with time in all groups (*P* < 0.05; Fig. 3A). At each time point, the γH2AX fluorescent intensity in the cells supplemented with NMN and NR was significantly lower than the control, but there was no difference between NMN and NR treatment groups (Fig. 3B). Similar results were obtained using the comet assay to assess DNA damage, in which both NMN- and NR-treated cells had shorter tail length than control cells (*P* < 0.05), while there was no difference between NMN and NR treated groups (Fig. 3C and D). These results indicated that NMN and NR promoted the repair of damaged DNA with similar efficacy.

### 3.4 NMN and NR restore intracellular NAD ^+^ levels in cisplatin-treated cells

NAD^+^ is important in maintaining cellular redox status, and cisplatin decreases intracellular NAD^+^ level [22]. It is still not clear whether there was a difference in the effect of NMN and NR in maintaining NAD^+^ levels in cisplatin-treated cells. Therefore, HeLa cells were treated as described above, and total NAD^+^ content was measured.

**Fig. 4:**
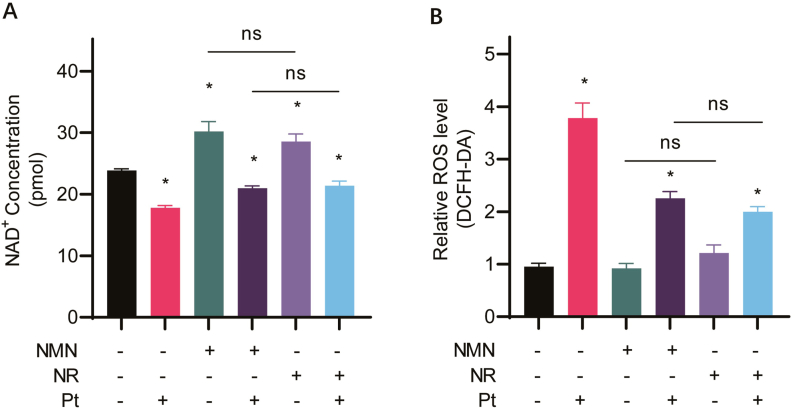
NMN and NR increase intracellular NAD ^+^ levels and decrease ROS levels in cisplatin-treated cells. HeLa cells were cultured in a medium containing 10 mmol/L NMN or NR with or without 10 μmol/L cisplatin (Pt) for 12 h, then (A) the intracellular NAD^+^ levels and (B) intracellular ROS levels were analyzed. *, *P* < 0.05, compared with untreated cells; ns, no significance.

As shown in Fig. 4A, compared with the untreated cells, cisplatin significantly decreased intracellular NAD^+^ levels (*Ρ* < 0.05), while both NMN and NR alone can increase cellular NAD^+^ levels (*Ρ* < 0.05). On the other hand, supplementation with NMN or NR restored NAD^+^ level in cells treated with cisplatin. However, there was no significant difference between NMN and NR for their effects on increasing intracellular NAD^+^ levels.

### 3.5 NMN and NR decreased cisplatin-induced intracellular ROS

As shown in Fig. 4B, compared to the control group, neither NMN nor NR alone affected cellular ROS levels, while the ROS levels in cisplatin-treated cells were significantly increased (*Ρ* < 0.05). However, in cisplatin-treated cells supplemented with NMN or NR, intracellular ROS level was decreased significantly, although it was still higher than that in the untreated control group (*P* < 0.05). There was also no significant difference in ROS levels between NMN/cisplatin and NR/cisplatin co-treatment groups, suggesting that NMN and NR have similar effects on reducing intracellular ROS levels.

## 4 Discussion

Maintaining intracellular NAD^+^ levels has been gradually recognized as an important approach for combating aging [23]. However, as NAD^+^ cannot be absorbed directly by cells, supplementation of NAD^+^ precursors, such as NMN and NR, which are presented in natural foods, including broccoli, tomatoes, milk, etc., has been tested in cell/animal models, as well as human clinical trials with relatively satisfying results [24]. Therefore, NMN and NR are now being marketed in many countries as food supplements. Nonetheless, whether these two compounds have the same efficacy is worth further investigation considering the huge market share and financial gain.

One key aspect of the beneficial effects of NMN and NR is the protection against DNA damage. Indeed, many studies have clearly demonstrated such effects. For example, a recent study showed that the administration of NMN maintained telomere length and dampened DDR by activating Sirt1 [8]. NMN administration could also significantly minimize tubular cell DNA damage and subsequent cellular senescence caused by H_2_O_2_ and hypoxia [25]. Similarly, NR reduced DNA damage levels in Alzheimer's disease model mice [9,26]. These results indicated that the NAD^+^ precursors, including both NMN and NR, could alleviate DNA damage, although a quantified comparison between the two has not been reported. In this study, interestingly, the results showed that NMN and NR mitigated cisplatin-induced DNA damage in a dose-dependent manner, but NR had a stronger protective effect than NMN (Fig. 2).

Based on the biosynthesis pathway for NAD^+^, NMN is a more direct precursor for NAD^+^, and thus should have better efficacy than NR. On the other hand, in mammals, exogenous NR is transported into cells through ENTs, while exogenous NMN has to be converted into NR by CD38 [14], and the conversion of NMN to NR is essential for it to act as an extracellular precursor of intracellular NAD^+^ in HEK293 cells [27]. NMN must be transformed into NR before intracellular transport, which may be the reason for its low efficiency of DNA damage protection. In contrast, it has also been reported that NMN can be directly transported into cells via a transporter coded by the SLC12A8 gene in the intestine of mice [28]. If this was the case, some other mechanisms should be investigated. However, as whether HeLa cells or human intestinal cells express SLC12A8 is not yet known, and the expression pattern of SLC12A8 in human tissues should be carefully examined. Nonetheless, in a recent study, it was reported that mice primarily rely on the nicotinamide and NR salvage pathways to generate NAD^+^ from NMN, while the uptake of intact NMN plays a minimal role [29]. If this is the case, then NR should have better efficacy than NMN.

One of the effects for cisplatin is the decreased cellular NAD^+^ levels (or decreased NAD^+^/NADH ratio) [30]. This imbalance in NAD^+^/NADH is coupled with the generation of excess ROS, which can lead to extensive DNA damage. Consequently, DDR is initiated by sensing the DNA damage, and various DNA repair systems are activated to repair the corresponding different types of DNA damage. Among them, NAD^+^ severed as an important substrate for the key DNA repair enzyme, PARP1 [31]. However, if intracellular NAD^+^ were exhausted, DNA repair might be interrupted, which eventually causes deleterious effects on cells, such as cell death. Therefore, the basis for the protective effects of NMN and NR lies in their ability to restore cellular NAD^+^ levels. Indeed, the results presented here showed that cisplatin decreased cellular NAD^+^ levels and increased ROS levels, while both NMN and NR could restore NAD^+^ levels, and reduced the ROS level of cisplatin-induced cells. However, there was no significant difference between the effects of NMN and NR.

Collectively, our results showed that both NMN and NR effectively protected HeLa cells against cisplatin-induced DNA damage. The two compounds have similar effects on enhancing cell viability, replenishing intracellular NAD^+^ and scavenging intracellular ROS. Interestingly, we found that NR might have a better protective (preventive) effect against cisplatin-induced DNA damage than NMN, which is a novel finding.

Still, there are some limitations for this study. For example, HeLa cells were used as the model system. Although it is probably the most commonly used cell line in laboratories across the world, as a cancer cell line, it may not reflect how normal cells would respond to NMN or NR. Another problem is that HeLa cells are known to be relatively resistant to cisplatin. This further hampered the application of the conclusion obtained from this study to other type of cells/tissues. To deal with such issues, currently we have conducted the same experiments using a normal cell line, the primary human hepatocytes (PHH), and obtained basically the same results (unpublished data). Thus, it appears that NMN and NR act indiscriminately regarding cell types. However, more detailed study using diverse cell types should be conducted to verify this conclusion. Also, the dosage of NMN and NR in the study was relatively high, and whether it is physiologically relevant is not clear. Currently, the supplementation dose for NMN or NR ranging from 150 mg to 1 g daily, which can be roughly converted to < 1 mmol/L, and thus is much lower than the doses in our study. Still, as NAD^+^ would function indiscriminately in both normal cell and cancer cell, our results implied that it may not be a good idea for cancer patients to take NMN or NR.

## Funding

This research was funded by the 10.13039/501100001809National Natural Science Foundation of China (Nos. 32270186, and 81772168), Zhejiang Provincial Natural Science Foundation (Nos. LY23H190001 and LQ18H190003), the Huadong Medicine Joint Funds of the 10.13039/501100004731Zhejiang Provincial Natural Science Foundation (No. LHDMZ24H040001), Hangzhou Bio-medicine and health industry development support science and technology project (Nos. 2021WJCY144, 2022WJC016) and Postgraduate research and innovation promotion program of Hangzhou Normal University (No. 1115B20500437).

## CRediT authorship contribution statement

**Shuting Qiu:** Writing – original draft, Methodology, Data curation. **Shihan Shao:** Writing – original draft, Methodology, Data curation. **Yunheng Zhang:** Writing – original draft, Methodology, Formal analysis, Data curation. **Yingying Zhang:** Methodology, Data curation. **Jie Yin:** Methodology, Data curation. **Yu Hong:** Validation, Methodology, Data curation. **Jun Yang:** Writing – review & editing, Investigation, Conceptualization. **Xiaohua Tan:** Writing – review & editing, Project administration, Investigation, Conceptualization. **Chunhong Di:** Writing – original draft, Project administration, Funding acquisition, Data curation.

## Declaration of competing interest

The authors declare that they have no known competing financial interests or personal relationships that could have appeared to influence the work reported in this paper.
